# From Baseline to Recovery: An Audit of Pre- and Post-ECT (Electro Convulsive Therapy) Patient Assessments

**DOI:** 10.1192/bjo.2025.10335

**Published:** 2025-06-20

**Authors:** Saba Ansari, Sujatha Maiya

**Affiliations:** Lanarkshire, Bellshill, United Kingdom

## Abstract

**Aims:** The audit was conducted to evaluate the effectiveness and procedural compliance of pre- and post-Electroconvulsive Therapy (ECT) assessments in accordance with the Scottish Electroconvulsive Therapy Accreditation Network (SEAN) Standard. Specifically, it assessed whether healthcare professionals are adhering to protocols by conducting the required Montreal Cognitive Assessment (MoCA), Montgomery–Åsberg Depression Rating Scale (MADRS), and Clinical Global Impression (CGI) evaluations. The goal is to enhance patient care and ensure strict adherence to established SEAN standards.

**Methods:** The audit utilized a retrospective analysis of patient records who received ECT in the two years 2022 and 2023. The focus was on the completeness of the MoCA, MADRS, and CGI assessments pre-ECT, immediately post-ECT, and during follow-ups at three and six months. ECT notes and digital notes were collected, and ECT packs were scrutinized to collect the data of the patients.

**Results:** Out of 32 patients evaluated, 20 underwent a Montreal Cognitive Assessment (MoCA) prior to electroconvulsive therapy (ECT). It was not feasible to conduct the assessment for 6 patients, and it remains not done for another 6. Post-ECT, only 10 patients have completed MoCA, with none receiving follow-up assessments at 3 or 6 months. Regarding the MADRS (Montgomery–Åsberg Depression Rating Scale), 28 patients were assessed before ECT. Two were unable to undergo this assessment, and it was not performed for another 2 patients. Post-ECT, 11 patients have completed their MADRS, but no follow-ups have been conducted at 3 or 6 months. For the Clinical Global Impression (CGI) scale, assessments were completed pre-ECT for 26 patients, with 2 unable to participate. Post-ECT, the CGI was completed for 13 patients, but there has been no follow-up at 3 or 6 months.

**Conclusion:** The audit reveals that while the pre-ECT MOCA, MADRS, and CGI assessments met established standards, there were notable gaps in the completion of post-ECT evaluations. Particularly concerning was the poor completion rate of these assessments at both the 3-month and 6-month intervals. To address this, the audit recommends implementing robust processes to ensure the consistent and timely completion of these crucial assessments, which are essential for evaluating not only the therapeutic efficacy of ECT but also its cognitive side effects. Additionally, the audit suggests the establishment of specialized clinics staffed by senior-level specialist nurses to conduct these assessments. This approach would not only facilitate the collection of comprehensive data on the effectiveness of ECT but also enhance research into its cognitive aspects.

